# Hospitals and State Aid.—II

**Published:** 1911-06-24

**Authors:** 


### HOSPITALS AND STATE INSURANCE.

### HOSPITALS AND STATE AID.—II.

CONDITIONS UNDER WHICH GOVERNMENT AID IS GIVEN TO HOSPITALS.

(BY OUR SPECIAL COMMISSIONER.)

### NEW ZEALAND.

THE interesting "Report on Hospitals and Charitable Aid in the Dominion, by the Inspector-General of Hospitals and Charitable Institutions," which, in pursuance of section 76 of the Hospitals and Charitable Institutions Act of 1909 is annually presented to both Houses of the New Zealand Legislature, contains much instructive reading. Not only does it show that the hospitals have made excellent progress during the year ending March 31, 1910—the limit fixed by the Act for the compilation of the yearly report—but it proves that the new Act itself, which came in for a good deal of criticism some time ago, is working satisfactorily. As the Inspector-General remarks, "The new Act is working well, and there is every reason for believing that it is an improvement on the old law. The personnel of the new Boards is good, and members are disposed to work harmoniously with the department."

### PUBLIC INTEREST IN HOSPITALS.

This in itself is encouraging. It is always an experiment fraught with a certain amount of risk to introduce into a system which is necessarily founded on such conservative principles as the voluntary hospital system is, a novel element that is open to the objection that it may tend, in course of time, radically to change the nature of these institutions. The objections to the law in New Zealand are in many ways interesting to us on this side, for they are closely analogous to the objections raised against any State or municipal interference in the management of English hospitals. It is, of course, quite true that conditions are not similar in the two countries. Necessarily, as we recently pointed out in these columns, lay interest in the management of hospitals is, at present at least, not so active or so expert in the Colonies as it is in this country. And when we use the expression lay we mean, of course, not a dilettante interest but the active interest taken in such hospital affairs by men who are not medical men or who have not had a special hospital training. It is fortunately true that the number of individuals, apart from these two latter classes, who are beginning to concern themselves seriously with hospital work in our self-governing Dominions is on the increase, and in the course of the next few years we will no doubt see arise in these States a keenly interested nucleus of public-spirited men who will devote a great deal of their time to personal service in the interests of charitable institutions. But at present we have no such body of men in existence, and it is no unfair criticism of the hospital system in the oversea States to say that their management and control are largely in the hands of, comparatively speaking, inexperienced lay committees. In the larger cities of Canada, Australia, and New Zealand this is not so much the case, but taken in general we think that we may with some justification claim to have a more eager public well-informed interest in hospital work in this country than exists at the present moment in the Colonies.

The situation in New Zealand is therefore an interesting one, for there can be little doubt but that the Act of 1909 will tend to promote this public interest as much as it is possible by legislative interference to do so. The work of the Inspector-General and the publication every year of such an able and instructive report as the one that lies before us, must inevitably encourage the spread of such interest.

### THE HOSPITALS ACT IN NEW ZEALAND.

Even more than is the case with the Commonwealth of Australia, the hospital system in the Dominion of New Zealand may claim to be an attempt to reach such perfection as is possible in a State where the population is as yet small, and where the demands upon the larger charitable institutions are not comparable to those which the hospitals in the older countries have to meet. The evolution of that system has been slow and gradual. Modelled at first entirely upon the institutions in the home country, the New Zealand hospitals, while voluntary public hospitals in the best sense of the term, were largely dependent on the Government for upkeep and maintenance. The voluntary principle was recognised in the freely offered personal service rendered by the staff of such an institution as a New Zealand public hospital, but it was felt that the comparative smallness of the population did not warrant the withdrawal of such aid from the hospitals as the Government was inclined to give. But here too it was recognised that State aid means a certain quid pro quo. The Government could not be expected to give such aid entirely without demanding in return the right to inspect the hospital from time to time, and of exercising a kind of paternal supervision over a system which, while inherently voluntary, nevertheless was admittedly, as yet, unable to depend for its existence entirely upon the charitable benefactions of the better-class section of the community. "Prior to the abolition of the provinces the Dominion hospitals were supported mainly out of State—that is, provincial—revenues. After that event the expenditure of the hospitals was for the most part charged against the revenues of counties and municipal corporations until October 1885, when the Hospitals and Charitable Institutions Act, 1885, was passed and came into force. Under this Act that portion of New Zealand included within the three principal islands—the North, South, and Stewart Islands—was divided into thirty-six hospital districts, each consisting of one or more counties with the interior boroughs and certain town districts having a population of 500 or more, presided over by thirteen hospital boards, twenty-three combined hospital and charitable aid boards, and six charitable-aid boards." *

This Act remained in force for more than twelve years, and worked fairly well, but it was felt that certain modifi-

*The Official Year Book for New Zealand. Published by Authority. 1910. To this interesting summary of the hospitals in the Dominion we are indebted for most of the information contained in this article.

cations and improvements were desirable, and after consultation with the hospitals and charitable associations an attempt was made to deal with the whole subject in an Act which is in many ways a model of what a hospital Act should be.

This is the Hospitals and Charitable Institutions Act of 1908, to consolidate and amend the laws relating to public hospitals and charitable institutions, the distribution of charitable aid, and the establishment of private hospitals. It repeals all former enactments dealing with the hospitals, and treats fairly and broadly the whole matter of charitable sick relief within the borders of the Dominion. By this Act the following system has been called into being.

The distinction between hospital boards and charitable-aid boards, as defined in the Act of 1885, has been abolished. Every hospital board now possesses the double function of maintaining hospitals and administering charitable relief in the district where it is located. Each board consists of representatives of the various contributory local districts, counties, boroughs, town districts, and in some instances road districts, as the case may be, lying within what is called a "hospital district." Representation on the board is proportionate to the population and rateable value of property in the contributory district, but the total number of members of any board is not to exceed twenty and is not to be less than eight. The representatives are chosen by the electors of the local authority of the contributory district, casual vacancies being filled by direct nomination by the local authority itself. Small contributory districts may be combined and return a representative in common. The representatives of each district retire at every general election of the local authority of that district, and their places are filled at an election held at the same time as the general election for the local authority. This arrangement preserves the continuity of the boards by securing the retirement of groups of members at different times, and also avoids the expense of separate and special elections. all property in connection with the purposes of the Act, including hospitals for infectious diseases, is vested in the boards, with the specific exception of certain public hospitals, four in number, and certain charitable institutions, six in number, which are specifically exempted under Act of Parliament, on the ground that they are entirely self-supporting so far as the local authorities are concerned. The revenues of the boards accrue from the following sources :—

(1) Rents and profits of lands and endowments.

(2) Voluntary contributions and bequests.

(3) Contributions from local authorities.

(4) Subsidies by Government from the Consolidated Fund, made under the following conditions : (*a*) Ten shillings in the pound on all devises or bequests, provided that this subsidy shall not exceed £500 in respect of the estate of any single testator; (*b*) twenty-four shillings in the pound on voluntary contributions other than bequests; (*c*) pound for pound on contributions by local authorities in respect of capital expenditure and in respect of other expenditure on a sliding scale, the amounts ranging from 12s. 3d. in the pound when the capital rateable value of property in the district exceeds 450 dollars per head of the population and the rate of levy per head of population is not under 4s., to 24s. 3d. when the capital rateable value of property per head of population is less than £100 and the levy per head is less than 2s.

The amount to be contributed by the local authority is determined by estimating the expenditure for the ensuing year, including any deficiency brought forward, and deducting from the amount so ascertained the probable revenue from all sources, excepting contributions from local authorities. The balance must be provided by the contributory bodies, either out of their ordinary revenues or by special rates. Should any body fail to pay the required contribution, the amount may be deducted from any subsidy or grant payable by the Government to the said local authority. The revenues of the boards are thus secured absolutely. When funds are required for the purpose of acquiring land as a site for any building, or for erecting, adding to, or altering any existing building, the contributory authority may raise the amount so required by way of a special loan from the Government Advances Board, repaying such loan by instalments.

Under specific clauses of the Act the contributory authority may demand an inquiry if it considers the proposed expenditure is unnecessary or extravagant, and if such contention is upheld an amended estimate and a fresh apportionment must be made.

All New Zealand hospitals under this Act are placed under the general supervision of the Ministry of Public Health. There is a special Minister in charge of hospitals and charitable aid (the Hon. George Fowldes, Minister of Education). At the head of the department specially commissioned to deal with hospitals is an inspector-general of hospitals (Dr. Valentine), who has under him three assistant inspectors (Miss Maclean, Miss Bickwell, and Miss Bayley). Mental hospitals are inspected by a special inspector-general of mental hospitals (Dr. Hay), who is aided by an assistant inspector of mental hospitals, Miss Maclean. The Act gives large powers to these inspectors. The inspector-general, for example, acts as general administrator of the Act under the direction of the Minister of Public Health, and is entrusted with plenary authority to inspect any institution within the meaning of the Act at any time he may think fit. If a hospital board fails or refuses to perform any duty imposed upon it by the Act, the inspector-general may, by special direction of the Minister in charge, take the matter into his own hands and remedy the defects, charging any expense incurred in so doing to the board. The Act makes provision for the control of private as well as public hospitals. For instance, it clearly lays down that, except in cases of emergency, the hours of employment of any nurse, probationer, attendant, or dresser in any hospital shall not exceed fifty-six in any one week. Private hospitals, which are defined as any institutions which are owned and controlled by private individuals for the care or relief of the sick or for the admission of maternity cases, were dealt with by the Private Hospitals Act of 1907. This Act has now been repealed, and its provisions incorporated in the Hospitals and Charitable Institutions Act of 1908, which provides for the licensing, management, and inspection of all private hospitals. Such institutions under the Act must be licensed, and every application for a licence must be accompanied by a statement giving a full description of the house or building which it is proposed to use, of the number of patients accommodated, and of the class of patients which it is proposed to receive. The licence shall state whether it is in respect of a lying-in private hospital, or a surgical and medical private hospital, or if for both classes of cases, and no private hospital shall be used for any purpose other than that in respect of which a licence is granted and purposes reasonably incidental thereto. Every private hospital must have a resident manager, who must be either the licensee, or some other duly approved person appointed by the licensee. In every case the manager must be a legally qualified medical practitioner or a registered nurse, in the case of a surgical and medical private hospital; or a registered midwife in the case of a private lying-in hospital; or a registered nurse and midwife, or a registered nurse having as resident assistant a registered midwife, in the case of a hospital licensed for both purposes. No licence shall be granted until the house and annexed buildings have been duly approved by the inspector-general of hospitals, and no addition shall be made to any private hospital until it has been so approved. No licence shall be granted until the character and fitness of the applicant have been proved, and every licence must be renewed annually. A proper register must be kept of all patients, showing names, address, age, date of reception, of leaving or death, name of medical attendant, and other details. Inquiry may be made at any time with regard to the management and conduct of any such private hospital, and if such inquiry prove unsatisfactory the licence may be revoked, and no new licence shall be granted to a person whose licence has been so revoked for a period not less than five years. Provision is made for the visitation and inspection of all private hospitals in the same manner as for the public institutions, and penalties are prescribed for breach of the regulations.

The Dominion of New Zealand, under this Act, is divided into thirty-six hospitals and charitable-aid districts, each containing one or more local districts, *i.e.* boroughs or counties with any subdivisions of the latter. The following table gives the latest available statistics with regard to the Dominion hospitals.

PUBLIC HOSPITALS IN NEW ZEALAND.

| | 1908-9 | 1909-10 |
|---|---|---|
| Number of institutions ... | 54 ... | 56 |
| Stipendiary medical staff ... | 73 ... | 80 |
| Nursing staff : | | |
| Trained nurses ... ... | 185 ... | 210 |
| Probationers ... ... ... | 436 ... | 452 |
| Domestic staff ... ... ... | 415 ... | 437 |
| Number of available beds ... | 2,502 ... | 2,689 |

THE REVENUE OF NEW ZEALAND HOSPITALS.

| | Year ending March 31. 1909 | 1910 |
|---|---|---|
| Government subsidies and grants | £100,255 | £89,007 |
| Contributions by local authorities... | £73,714 | £82,094 |
| Voluntary contributions ... ... | £17,110 | £20,752 |
| Patients' payments ... ... | £32,382 | £37,541 |
| Other receipts ... ... ... | £24,321 | £14,494 |
| Total | £247,782 | £243,888 |

The amounts contributed by the general Government and by the local authorities represent 3s. 8d. per head of the population in 1908-9, and 3s. 6¾d. per head of population in 1909-10.

There is as yet no invalidity insurance in the Dominion. Under the Act of 1901, which provides for the payment of accident insurance by Government, provision is made for the payment of certain rates of pay to individuals insured who may have contracted certain occupation diseases such as anthrax, chronic lead poisoning, etc., such diseases being regarded as accidents within the meaning of the Act. The payments are limited to six years, and cannot exceed £500 in any case, reckoned continuously. Up to the present it does not appear that this Act has had any appreciable effect upon the hospitals.

Some interesting details of the Act and its provisions are given in Dr. Valentine's report, already quoted. He outlines the clauses of the Act as follows :—

1. Election of members of Boards supersedes the previous system of nomination. A Hospital Board consists, as before, of representatives of the various contributory districts within the hospital district, the representation on the Board being proportionate to the population and the value of the rateable property in the contributory district. The representatives are, however, elected by the electors of the local authority of the contributory districts instead of being nominated by the local authorities themselves, who, however, have the power to fill casual vacancies. Small contributory districts may, as before, be combined together by the Governor and return a representative in common.

2. Continuity of office : Representatives elected at the first election of Boards under the Act will hold office for two years certain. Thereafter the representatives of any contributory district will retire at every general election of the local authority of that district, and their places will be filled by an election held at the same time as that general election. This arrangement preserves the continuity of the Boards by securing the retirement of groups of members at different times, and also prevents the necessity of any separate and special election by using the existing machinery of local elections. Provision is made for the Chairman of the Board to hold office for two years.

3. One body administers both hospitals and charitable aid in its district : The distinction between Hospital Boards and Charitable-Aid Boards is abolished, and every Board possesses the double function of maintaining hospitals and administering charitable relief.

4. All existing separate institutions (except a few which are specifically exempted on the ground that they are self-supporting so far as the local authorities are concerned), are vested in the Hospital Boards of the districts in which they are situated, and they cease accordingly to exist as separate institutions.

5. No new separate institution can be established. Charitable bodies desiring incorporation may, as the law now stands, register under the Unclassified Societies Act.

6. The existing separate institutions which are not transferred to the Boards retain their present corporate existence and independent management and their right to Government subsidies, but have no right to obtain financial assistance from the Hospital Board or the local authorities.

7. Every institution under the control of a Board is to be directly administered by a committee nominated by the Board. The committee may comprise persons who are not members of the Board. The powers of the committees are completely subordinate to those of the Board.

8. Infectious-diseases hospitals are made subject in all respects to the same law as ordinary hospitals, except that the Chief Health Officer can, as at present, require sufficient provision for infectious diseases to be made.

9. Under the repealed Act in two of the large centres no less than four different bodies administered hospitals and charitable aid, the functions of two of the Boards being simply to find the money, in the expenditure of which they had practically no voice.

10. Government subsidies : These remain as before as regards the 24s. in the pound granted on voluntary contributions and the 10s. in the pound granted on bequests. With a view, however, of helping poor districts, and at the same time penalising extravagant administration, the previous subsidy of £1 for £1 on the amount collected by levies on local authorities is withdrawn, and subsidy is granted on the basis shown in the table on p. 320.

It will thus be seen that a poor district with a low rateable value per head gets a higher rate of subsidy than a rich district, whose high rateable value also presupposes a less number of poor to be provided for, and if such rich district is extravagant in its expenditure and has to levy at a high rate per head of its population, it receives a still lower subsidy. The subsidy of £1 for £1 on loans for capital expenditure, however, remains as before.

*Rates of Subsidy for each Pound of Contributions levied under Contributory Local Authorities.*

| First Column | Second Column | | | | | |
|---|---|---|---|---|---|---|
| | Rate of Levy per Head of the Population | | | | | |
| Rateable Value per Head of the Population | Under 2s. | Under 2s. 6d., but not under 2s. | Under 3s., but not under 2s. 6d. | Under 3s. 6d., but not under 3s. | Under 4s., but not under 3s. 6d. | Not under 4s. |
| | s. d. | s. d. | s. d. | s. d. | s. d. | s. d. |
| Under— £100 ... ... ... | 24 3 | 24 0 | 23 9 | 23 3 | 22 3 | 20 3 |
| £150 but not under £100 | 23 3 | 23 0 | 22 9 | 22 3 | 21 3 | 19 3 |
| £200 but not under £150 | 22 3 | 22 0 | 21 9 | 21 3 | 20 3 | 18 3 |
| £250 but not under £200 | 21 3 | 21 0 | 20 9 | 20 3 | 19 3 | 17 3 |
| £300 but not under £250 | 20 3 | 20 0 | 19 9 | 19 3 | 18 3 | 16 3 |
| £350 but not under £300 | 19 3 | 19 0 | 18 9 | 18 3 | 17 3 | 15 3 |
| £400 but not under £350 | 18 3 | 18 0 | 17 9 | 17 3 | 16 3 | 14 3 |
| £450 but not under £400 | 17 3 | 17 0 | 16 9 | 16 3 | 15 3 | 13 3 |
| Not under £450 ... | 16 3 | 16 0 | 15 9 | 15 3 | 14 3 | 12 3 |

11. Better provision is made for the very difficult matters of relief afforded by one Board to residents in the district of another.

12. A certain degree of Ministerial control is given in respect of medical and other appointments by Boards, the framing of bylaws, and expenditure on new buildings.

13. Two or more Boards may by agreement combine together to establish any hospital, sanatorium, or other institution, to be managed by a joint committee.

14. Boards' powers are extended to embrace public health: A Board may, with the consent of the local authorities in its district, be declared the local authority of such district for the purpose of the Public Health Act.

Commenting on the working of this measure Dr. Valentine writes:—

A few local authorities neglected to hold elections, and their representatives on the Board were subsequently appointed by his Excellency.

Many members of the old Boards were elected, consequently the personnel of the elected Board differ very little from that of Boards nominated under the old *régime*—a point which, at this particular stage of social development in this country, may be viewed with considerable satisfaction. In fact, there was a distinct disposition on the part of the newly elected Boards to avail themselves of the services of those who had seen the gradual development of our hospital system, and to appoint, as co-operative members of committee, persons who were recognised to have done good service under the old law, but who for one reason or another were disinclined to allow themselves to be nominated for election.

The new Boards have settled down to their work with a good deal of zeal. In many districts committees with limited powers have been appointed to carry out the details of the Boards' work, particularly with regard to institutional management and outdoor relief. As time goes on, the tendency will undoubtedly be to extend the powers of these committees. This must be carefully watched.

It is early yet to expect the Boards to recognise the significance of the new law, and extend its operations into wider fields. The machinery is available, but let it rest until the base of operations is made secure, for not until then should the general advance be sounded.

Dr. Valentine suggests the following subjects which should engage the attention of the Boards during the coming year:—

1. *The fixing of the Base of Operations.*—(*a*) This should be the largest hospital of the district, and the Board's offices should be as handy to this hospital as possible. By this means the cost of administration should be lessened, as it would tend to prevent reduplication of the clerical staff. The Board's secretary would be in touch with the various executive officers, and therefore better able to supervise the conduct of the institution. (*b*) From the parent or base hospital patients could be drafted to suitable outlying institutions—the chronic ward, the Old People's Home, the Convalescent Home, etc. (*c*) From the parent hospital could also be drafted nurses to staff such outlying institutions, including the small country hospitals. By this means their training would be more varied, and would better qualify them for administrative work. It would enable nurses, who would otherwise have to be trained in country hospitals, to obtain the "mana" of having been trained in a large hospital. The larger Boards are now working in this direction.

2. *Self-contained Districts.*—The larger hospital districts should be self-contained—*i.e.* should be in a position to provide accommodation for all classes of the sick and needy in suitable parts of the district, namely: (*a*) The acutely sick, including—i. The sick infant; ii. The mentally defective (awaiting examination); iii. The delirium tremens patient; iv. The venereal patient (classes that have been hitherto somewhat neglected. (*b*) The chronic and incurable. (*c*) The "infectious" patient. (*d*) The consumptive—i. Curable; ii. Incurable (a special branch of the out-patient department might be devoted to encouraging persons in the pre-phthisical stages to seek advice. Such measures, combined with a system of district nursing, might do good work in the fight against this disease). (*e*) The maternity patient—i. By outside medical attendance; ii. By district (midwife) nurses; iii. By maternity wards attached to certain hospitals. (*f*) The aged and needy—i. Indoor and outdoor relief. (*g*) The out-patient, by means of a special department attached to the hospital in conjunction with a system of district nursing. A district nurse in touch with an out-patient department could do great work in visiting those persons who are receiving treatment as out-patients, or who have recently been discharged from the hospital, in seeing that they are conforming to the treatment prescribed, and that they are living under sanitary conditions.

3. *Public Health.*—It would greatly facilitate the work of the Boards, and entail an economical administration, if they assumed the responsibilities of a local authority under the Public Health Act, as they are entitled to do under Section 83 of the Hospitals Act, especially with regard to the control of infectious diseases.

All Hospital Boards, and almost all local authorities interviewed on this subject, have confirmed the principle that the authority responsible for the accommodation of the sick should be also responsible for those influences that are likely to cause or spread sickness. In some districts the local authorities have agreed to give up all their powers under the Public Health Act to the Hospital Boards, and the latter have cheerfully accepted the additional responsibility.

4. *Co-operation between Public and Private Philanthropy.*—It should be the aim of all Boards to co-operate with private societies, with a view to prevent the overlapping that is now going on.

A great deal could be done in this direction if those concerned would but approach the matter in an impartial spirit, and waive all local or sectarian prejudices.

A number of private philanthropic societies are doing good work in the country, but, as they are not in touch with each other—indeed, some are working in open antagonism—nor with the Hospital Boards, many undeserving persons are receiving relief which would not be given if there were some system of organisation.

It would be quite proper for the Boards to give pecuniary assistance to those private societies which are doing good work that would otherwise have to be undertaken by the Boards, and by giving such assistance the Government subsidies would be given automatically. The Boards could allow societies thus subsidised to manage their own affairs, either as co-operative members of committees that Boards are entitled to set up, or altogether free of the Boards, provided the latter were made aware as to the exact ground each society was covering, so that executive officers could work in unison.

Dealing with the report itself, we note that the total receipts for the year 1909-10 were £276,805, as against £28,379 during 1908, the amount from patients' payments accounted for £4,574, as against £28,397 during 1908, while the amount from voluntary contributions shows a slight decrease. Roughly speaking, one-third of the hospital receipts are derived from the rates, one-third from the Consolidated Fund, and one-third from patients' payments, voluntary contributions, and other sources. The total cost per bed, averaging all the hospitals, shows a decrease of nearly £7, being now £100 7s., as against £107 0s. 1d. in 1908. With reference to the figures quoted under the various headings under expenditure, the tables are of great interest to those who like to study hospital economics. Under the heading Surgery and Dispensary, Dr. Valentine writes :—

The drug and dressing account for the Dominion is still stupendous. Though Hospital Boards have been circularised as to the reasonable cost of articles in daily use, and asked to submit tenders, there has not been the response that was hoped for. For fear of offending the local chemist, some authorities continue to pay highly—at times double the prices paid by others.

We must not be content with a small decrease in this item, and I fear that until the Department can initiate a bureau for the purchase and distribution of drugs and dressings the present high prices will rule.

New Zealand has at present 2,689 hospital beds, at which patients are treated at an average cost of 5s. 7d. per day, of which 1s. 2d. is paid by patients themselves, so that the cost to the taxpayer is 4s. 5d. per day. The gross cost of hospitals is 4s. 10¼d. per head of population, of charitable aid 2s. 3d., or altogether 7s. 1¼d. These figures are on the whole very satisfactory and show an improvement on those of the past year, especially when one bears in mind that owing to the necessary march of science and the increased cost of treatment, the net cost of treatment has risen considerably.

We have, unfortunately, no space to deal at length with the many other interesting points in this report —the study of which we commend to every one who is interested in hospital progress.

